# A New Technique for Quantitative Determination of Dexamethasone in Pharmaceutical and Biological Samples Using Kinetic Spectrophotometric Method

**DOI:** 10.1155/2015/439271

**Published:** 2015-02-08

**Authors:** Ali Mohammad Akhoundi-Khalafi, Masoud Reza Shishehbore

**Affiliations:** Department of Chemistry, Yazd Branch, Islamic Azad University, Yazd 89195-155, Iran

## Abstract

Dexamethasone is a type of steroidal medications that is prescribed in many cases. In this study, a new reaction system using kinetic spectrophotometric method for quantitative determination of dexamethasone is proposed. The method is based on the catalytic effect of dexamethasone on the oxidation of Orange G by bromate in acidic media. The change in absorbance as a criterion of the oxidation reaction progress was followed spectrophotometrically. To obtain the maximum sensitivity, the effective reaction variables were optimized. Under optimized experimental conditions, calibration graph was linear over the range 0.2–54.0 mg L^−1^. The calculated detection limit (3*s*_b_/*m*) was 0.14 mg L^−1^ for six replicate determinations of blank signal. The interfering effect of various species was also investigated. The present method was successfully applied for the determination of dexamethasone in pharmaceutical and biological samples satisfactorily.

## 1. Introduction

**Scheme 1:**
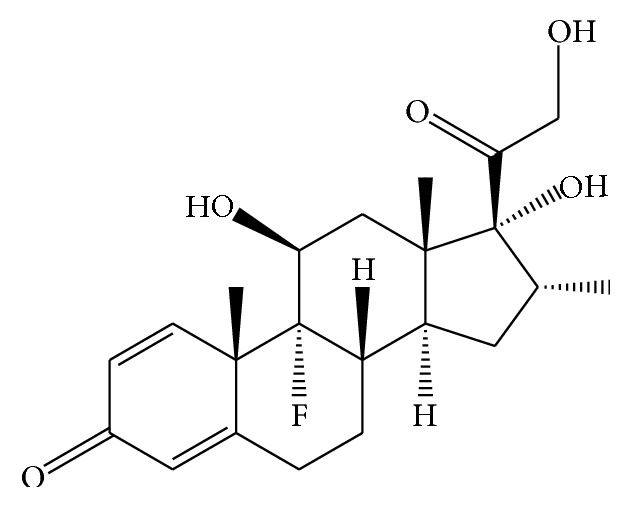
Molecular structure of dexamethasone.

Dexamethasone, see Scheme 1 for molecular structure, is a synthetic member of the glucocorticoid class of steroid drugs that has anti-inflammatory and immunosuppressant effects. It is more potent than cortisol in its glucocorticoid effect, while having minimal mineralocorticoid effect. Based on the WHO's list of Essential Medicines, dexamethasone is of the most important medications needed in a basic health system [1]. It is used to treat many inflammatory and autoimmune conditions, such as rheumatoid arthritis and bronchospasm [2, 3]. In addition, it is given in small amounts before and/or after some forms of dental surgery and useful to counteract allergic anaphylactic shock, if given in high doses. It is present in certain eye drops and as a nasal spray and certain ear drops [4, 5]. With respect to the widespread use of the drug, developing the rapid, low cost, and reliable procedure for quantitation of it in real samples with different matrices is necessary.

Various reports have been found for quantitative determination of dexamethasone in real samples with different matrices. They are including spectrophotometry [6], liquid chromatography [7, 8], liquid chromatography-mass spectrometry [9–11], and electrochemical methods [12]. The methods have limitations such as high cost and hard operation [7–11] and low repeatability [12].

Kinetic spectrophotometric method that has advantages such as high sensitivity, sufficient accuracy, simplicity, speed, and the necessity of less expensive apparatus makes it as an attractive method for the determination of trace elements in samples with different matrices such as foods [13] and biological and pharmaceutical [14, 15] samples.

In continuing of our research interest for the determination of drugs, a simple, rapid, selective, and sensitive kinetic spectrophotometric method was developed. The method is based on the catalytic effect of dexamethasone on the Orange G-bromate reaction system. The developed method has been successfully applied for the determination of dexamethasone in pharmaceutical and biological samples. To the best of our knowledge, we do not have any report for the determination of dexamethasone using kinetic spectrophotometric method.

## 2. Experimental

### 2.1. Apparatus

A single beam Agilent UV-Vis spectrophotometer (8453, USA) with 1 cm glass cell was used to measure the absorbance. A thermostated water bath (Heidolph, Germany) was used to keep the temperature of all solutions at the working temperature (25.0 ± 0.1°C). A stopwatch was used to record the reaction time.

### 2.2. Chemicals

Doubly distilled water and analytical reagent grade chemicals were used. Dexamethasone as dexamethasone phosphate (Sigma) stock solution 100.0 mg L^−1^ was prepared just before use by dissolving appropriate amount of dexamethasone phosphate in water and diluted to the mark in a 100 mL calibrated flask. Working solution was prepared by serial dilution. A solution of Orange G (6.6 × 10^−4^ mol L^−1^) was prepared by dissolving 0.2985 g of Orange G (Merck) in water and diluting to 1.0 L with water. Sulfuric acid solution (4.0 mol L^−1^) was prepared by appropriate dilution of conc. sulfuric acid (Merck). A 0.05 mol L^−1^ of potassium bromate solution was prepared by dissolving 8.3504 g of KBrO_3_ (Merck) in water and diluting to 1.0 mL in a calibrated flask.

### 2.3. Recommended Procedure

The catalyzed reaction was studied spectrophotometrically by monitoring the change in absorbance of the reaction mixture at 478.5 nm (*λ*_max⁡_). For this purpose, to a series of 10 mL volumetric flasks, 0.8 mL of 6.6 × 10^−4^ mol L^−1^ of Orange G solution, 1.5 mL of 4.0 mol L^−1^ sulfuric acid solution, and the standard solutions containing 0.02–0.54 mg of dexamethasone were added. The solution was mixed and diluted to 8 mL with water. Then, 1.0 mL of 0.05 mol L^−1^ of bromate solution was added and diluted to the mark. A time measurement was started just after adding the last drop of the bromate solution. After thorough mixing, a portion of the solution was transferred to a glass cell. The absorbance of catalysed reaction (Δ*A*_s_) was measured against water at *λ*_max⁡_ and 20°C for time interval 30–420 s. The measurement in the absence of dexamethasone was repeated to obtain the values for the uncatalysed reaction (Δ*A*_b_). Finally, the difference in the absorbance change was considered as the response (Δ*A* = Δ*A*_s_ − Δ*A*_b_). The calibration graph was constructed by plotting the response against the dexamethasone concentration.

### 2.4. Procedure of Sample Preparation

#### 2.4.1. Pharmaceutical Samples Preparation

Ear-eye drop (0.1%) and injection solution (8.0 mg) were used as pharmaceutical samples. Each sample was diluted properly and appropriate amount of the solution was used in each analysis.

#### 2.4.2. Biological Samples Preparation

Dexamethasone was determined in human urine and serum as biological samples. They were spiked with dexamethasone and solid phase extraction technique with C_18_ cartridge (Supelco Inc., 10 mL) was used for purification and preconcentration of dexamethasone from the samples. The purification and preconcentration procedure was performed as discussed in [16]. According to the procedure, the extracted dexamethasone was determined.

## 3. Results and Discussion

Orange G, a yellowish synthetic azo-based dye, widely used as color marker for following the electrophoresis process, pH indicator, dyeing of textiles, paper, and leather, and preparing coloring inks [17]. It can be oxidized to a colorless product by oxidizing agents [18].

**Figure 1:**
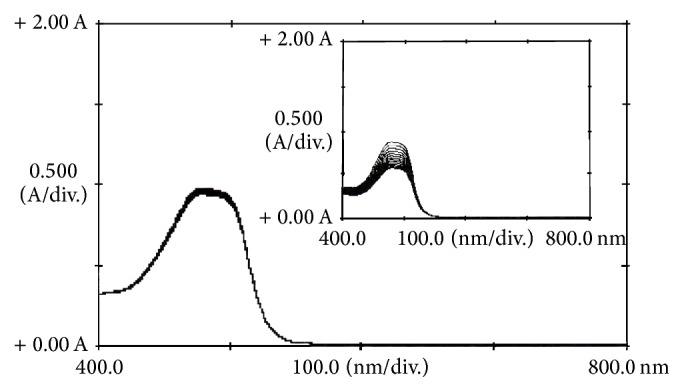
Absorption spectra of the uncatalysed reaction. (Conditions: Orange G, 52.8 *μ*mol L^−1^; sulfuric acid, 0.6 mol L^−1^; bromate, 5.0 mmol L^−1^; 25°C and 420 s). Inset shows the absorption spectra of the catalysed reaction (in presence of 0.5 mg L^−1^ of dexamethasone).

The capability of the reaction system (Orange G, sulfuric acid, and bromate) for the determination of dexamethasone was evaluated by following the change in absorbance in absence (Figure 1) and presence (inset of Figure 1) of it. Comparison of the two spectra indicated that trace amounts of dexamethasone can increase the reaction rate seriously. Therefore, the proposed reaction system can be used for the determination of dexamethasone.

The suggested reaction mechanism for proposed reaction system may be represented as follows.

The uncatalysed reactions that resulted in blank signal (Δ*A*_b_) was carry out in a cyclic way by these reactions:

(1) Orange  GRed+BrO3−+6H+⟶Orange  GOx  +Br−+3H2O

(2) $$\begin{array}{c} 6{{\text{BrO}}_{3}}^{-}+10{\text{H}}^{+}+12{\text{Br}}^{-}⟶6{\text{Br}}_{2}+6{\text{H}}_{2}\text{O} \end{array}$$

(3) $$\begin{array}{c} \text{Orange}\;{\text{G}}_{\left(\text{Red}\right)}+{\text{Br}}_{2}+{\text{H}}^{+}⟶\text{Orange}\;{\text{G}}_{\left(\text{Ox}\right)}+2{\text{Br}}^{-} \end{array}$$

In the presence of dexamethasone

(4) DexamethasoneRed+Br2+H+⟶2Br−+Dexamethasone(OX)

where Red and OX are the reduced and oxidized forms of the reactant, respectively.

Since dexamethasone along with the Orange G participates on Br^−^ generation, the possibility of Br_2_ generation was increased (reaction (2)) and resulted in increasing the possibility of decolorization of Orange G (reaction (3)). Therefore, the change in absorbance in presence of dexamethasone was increased dramatically.

### 3.1. Optimization of the Effective Factors

In order to obtain the maximum sensitivity as employing the proposed procedure, the effective factors including reagents concentration and reaction conditions must be optimized. The maximum response was considered to obtain the most sensitive results.

#### 3.1.1. The Effect of Orange G Concentration

**Figure 2:**
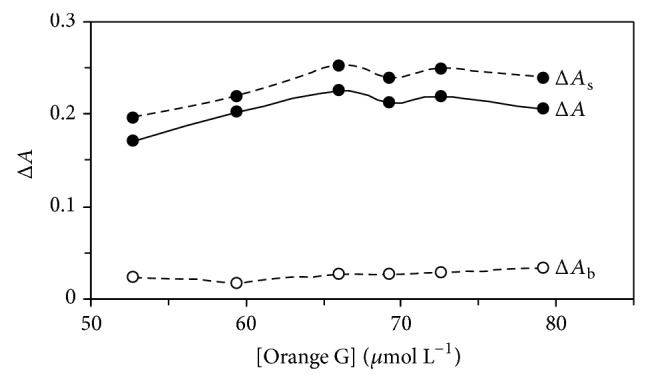
Effect of Orange G concentration on the rate of uncatalysed (Δ*A*_b_) and catalysed (Δ*A*_s_) reactions and response (Δ*A*). (Conditions: sulfuric acid, 0.6 mol L^−1^; dexamethasone, 0.5 mg L^−1^; bromate, 5.0 mmol L^−1^; 25°C and 420 s).

The effect of Orange G concentration on the rate of reaction was studied over the range 52.8–79.2 *μ*mol L^−1^. As it can be seen in Figure 2, the sensitivity was increased up to 66.0 *μ*mol L^−1^ of Orange G. At higher concentrations, the reaction rate was decreased which may be attributed to the dye aggregation. Thus, 66.0 *μ*mol L^−1^ of Orange G as optimum concentration was selected for further study.

#### 3.1.2. The Effect of Sulfuric Acid Concentration

**Figure 3:**
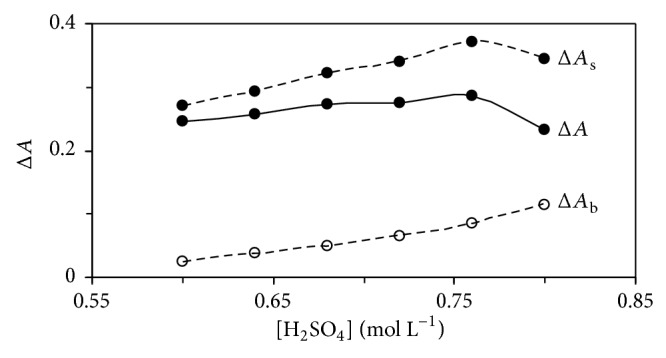
Effect of sulfuric acid concentration on the rate of uncatalysed (Δ*A*_b_) and catalysed (Δ*A*_s_) reactions and response (Δ*A*). (Conditions: Orange G, 72.6 *μ*mol L^−1^; dexamethasone, 0.5 mg L^−1^; bromate, 5.0 mmol L^−1^; 25°C and 420 s).

The effect of sulfuric acid concentration on the catalyzed and uncatalyzed reactions was studied over the range of 0.6 to 0.8 mmol L^−1^ (Figure 3). The maximum sensitivity was obtained at 0.76 mmol L^−1^, whereas at higher concentrations the sensitivity was decreased. Protonation of Orange G at higher acid concentrations that makes its oxidaion to be quite difficult resulted to the disorder. Therefore, 0.76 mol L^−1^ of sulfuric acid was used for further study.

#### 3.1.3. The Effect of Bromate Concentration

**Figure 4:**
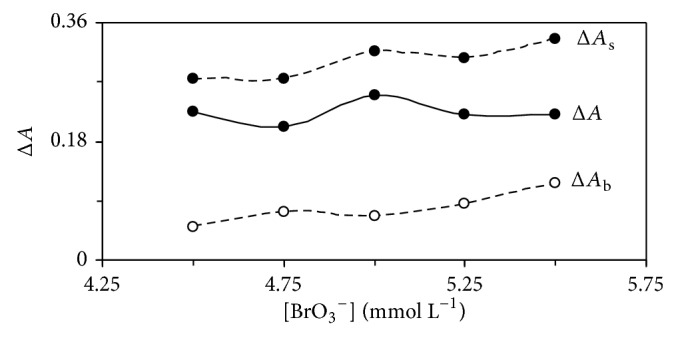
Effect of bromate concentration on the rate of uncatalysed (Δ*A*_b_), catalysed (Δ*A*_s_) reactions, and response (Δ*A*). (Conditions: Orange G, 72.6 *μ*mol L^−1^; sulfuric acid, 0.6 mmol L^−1^; dexamethasone, 0.5 mg L^−1^; 25°C and 420 s).

The effect of bromate concentration on the reaction rate was studied in concentration range 4.5–5.5 mmol L^−1^. As shown in Figure 4, the net reaction rate was increased up to 5.0 mmol L^−1^ which was selected as being the optimum concentration of oxidant.

#### 3.1.4. The Effect of Temperature

Under optimum experimental conditions, the effect of the temperature on the reaction rate was studied in the range of 15 to 45°C. Increasing the temperature up to 20°C caused an increase in the sensitivity, whereas at higher temperatures it decreased. Thus, 20°C was selected as being the optimum temperature.

#### 3.1.5. The Effect of Time

The optimum time was found by measuring the change in the absorbance during 30–540 s. The reaction rate increased up to 420 s, and in longer times the reaction rate was almost constant. Therefore, 420 s was selected for further study.

### 3.2. Analytical Parameters

#### 3.2.1. Linearity

Calibration graph was constructed by plotting the response against dexamethasone concentration. Using the recommended procedure and under optimized conditions that are outlined above, calibration graph was linear over the range 0.2–54.0 mg L^−1^ of dexamethasone including two segments of 0.2–12.0 and 12.0–54.0 mg L^−1^. The regression equation of the two segments gives (5) and (6), respectively.

(5) $$\begin{array}{c} \Delta A=0.0154\left[\text{dexamethasone}\right]+0.0071\;\left(R^{2}=0.9987\right), \end{array}$$

(6) $$\begin{array}{c} \Delta A=0.0032\left[\text{dexamethasone}\right]+0.1574\;\left(R^{2}=0.9984\right), \end{array}$$

where Δ*A* is the difference in the absorbance between the blank and the sample and [dexamethasone] is the dexamethasone concentration in mg L^−1^ and *R* ^2^ is the correlation coefficient.

#### 3.2.2. Limit of Detection (LOD) and Precision

The limit of detection (3*s*_b_/*m*; *s*_b_ is the standard deviation of the blank signal and *m* is the slope of calibration curve) was 0.14 mg L^−1^ of dexamethasone for six replicate determinations. The relative standard deviations (*n* = 6) were 2.92 and 2.23% for 0.5 and 5.0 mg L^−1^ of dexamethasone, respectively.

#### 3.2.3. Interference Studies

The interfering effect of foreign species on the determination of 2.0 mg L^−1^ of dexamethasone was investigated. The tolerance limit was defined as the concentration of the added species causing an error more than ±5% on analytical signal. The results are given in Table 1. The obtained results show that chloride and nitrite have seriously interfering effect, whereas they do not exist in real sample matrix. Also, the interfering effect of cortisol that has the same effect on dexamethasone and can be coexisting with the analyte in real samples matrices was investigated. The tolerance limit of 8.3 fold than 2.0 mg L^−1^ of dexamethasone which was not more than the upper limit of cortisol in blood confirms the proposed method is free from the cortisol interfering effect. Therefore, the presence of cortisol can not affect the determination of dexamethasone.

#### 3.2.4. Real Samples Analysis

Evaluating the reliability and analytical applicability of the developed method makes it potentially useful for the quantitative determination of dexamethasone in real samples with different matrices. Pharmaceutical sample preparation was performed as discussed previously (Section 2.4.1). An appropriate amount of the samples was analyzed by the recommended procedure. The results of three replicate determinations were given in Table 2. The precision (RSD%) is near 1% for both analyzed pharmaceutical samples. The obtained value confirms the repeatability of the developed method. The reliability of the method was evaluated by statistical *t* test. The experimental *t* values for eye-ear drop (1.72 and 1.73) and injection solution (1.74 and 0.88) are different from the critical value (4.30, 95% confidence level, and two degrees of freedom). Regarding the difference between the experimental and critical *t* values, the systematic error for the determination of dexamethasone in pharmaceutical samples using the developed method is negligible. Also, the procedure was used for the determination of dexamethasone in urine and serum samples. After sample preparation (Section 2.4.2) they were spiked with different amounts (2.0, 8.0, and 20.0 mg L^−1^) of dexamethasone and analysed using developed procedure. The obtained results were given in Table 3. The accuracy of the procedure was confirmed by recovery. The recoveries of the spiked urine and serum samples vary over the range 99.4–102.0% and 99.0–100.7%, respectively. The recovery values are near 100% and confirm that the systematic error during the quantitative determination of dexamethasone in biological samples is slight. Successive applications of developed method for drug determination in pharmaceutical preparations and urine samples were performed. Therefore, the developed method is free from interfering effect of matrix effect and suitable for analysis of dexamethasone in different samples.

## 4. Conclusion

This study reports a sensitive and relatively selective spectrophotometric method for the determination of dexamethasone using a new reaction system. The developed method possesses distinct advantages over other existing methods in cost, simplicity, ease of operation, and applicability to real samples analysis. Also, the reliability of the method permits the analysis of pharmaceutical and biological samples satisfactorily.

## Figures and Tables

**Table 1:** Tolerance limit for foreign species on the determination of 2.0 mgl L^−1^ of dexamethasone.

Foreign species	Tolerance limit (*W*_Dexamethasone_/*W*_Species_)
Saccarose, fructose, and glucose	1000
SO_4_^2−^, CH_3_CO_2_^−^, HCO_3_^−^, CO_3_^2−^	1000
NO_3_^−^	965
Ethanol	940
Urea	745
Uric acid	252
Cl^−^	11
Cortisol	8.3
NO_2_^−^	1

**Table 2:** Determination of dexamethasone in ear-eye drop (0.1%) and injection solution (8 mg/amp) using the developed procedure.

Sample	Found^a^	RSD (%)	Labeled	Statistical *t* test^b^	Pharmaceutical company/batch number
Ear-eye drop					
1	0.99 ± 0.001	1.01	0.1%	1.73	Daru Pakhsh-Iran/514
2	0.101 ± 0.001	0.99	0.1%	1.72	Daru Pakhsh-Iran/519
Injection solution					
1	7.92 ± 0.80	1.00	8	1.74	Daru Pakhsh-Iran/352
2	8.04 ± 0.79	0.98	8	0.88	Daru Pakhsh-Iran/309

^a^Mean ± standard deviation (*n* = 3).

^b^Tabulated *t*-value for two degrees of freedom at *P*(0.95) is 4.30.

**Table 3:** Determination of dexamethasone in human serum and urine samples using the developed procedure.

Sample	Added (mg L^−1^)	Found^a^ (mg L^−1^)	RSD (%)	Recovery (%)
Human urine				
1	—	<D.L	—	<D.L
	2.0	2.04 ± 0.02	1.03	102.0
	8.0	8.09 ± 0.08	1.01	101.1
	20.0	19.89 ± 0.20	1.00	99.4
Human serum				
1	—	<D.L	—	<D.L
	2.0	1.98 ± 0.02	1.01	99.0
	8.0	8.04 ± 0.08	0.99	100.5
	20.0	20.14 ± 0.10	0.94	100.7

^a^Mean ± standard deviation (*n* = 3).
